# ^1^H, ^13^C, ^15^N resonance assignment of the enzyme KdgF from *Bacteroides eggerthii*

**DOI:** 10.1007/s12104-022-10102-6

**Published:** 2022-08-30

**Authors:** Agnes Beenfeldt Petersen, Idd Andrea Christensen, Mette E. Rønne, Emil G. P. Stender, David Teze, Birte Svensson, Finn Lillelund Aachmann

**Affiliations:** 1 Department of Chemistry, DTU Technical University of Denmark, 2800 Kgs. Lyngby, Denmark; 2 NOBIPOL, Department of Biotechnology and Food Science, NTNU Norwegian University of Science and Technology, Trondheim, Norway; 3 Enzyme and Protein Chemistry, Department of Biotechnology and Biomedicine, DTU Technical University of Denmark, 2800 Kgs. Lyngby, Denmark

**Keywords:** KdgF, Alginate degradation, 4-deoxy-L-erythro-5-hexoseulose (DEH), Human gut microbiota

## Abstract

To fully utilize carbohydrates from seaweed biomass, the degradation of the family of polysaccharides known as alginates must be understood. A step in the degradation of alginate is the conversion of 4,5-unsaturated monouronates to 4-deoxy-L-erythro-5-hexoseulose catalysed by the enzyme KdgF. In this study *Be*KdgF from *Bacteroides eggerthii* from the human gut microbiota has been produced isotopically labelled in *Escherichia coli*. Here the ^1^H, ^13^C, and ^15^N NMR chemical shift assignment for *Be*KdgF is reported.

## Biological context

There is a growing demand for new biomass globally. A recent report by EIT Climate-KIC concluded that by 2050 the biomass usage in Europe is expected to increase 70−150%, which current biomass sources cannot sustain (Material Economics 2021). A currently underutilised biomass is seaweed. Seaweed is a fast-growing plant that does not require arable land, fresh water, or fertilizer (Enquist-Newman et al. 2014). Seaweed therefore has the potential to be a sustainable source of biomass e.g. for the production of chemical commodities, like biofuels. Alginate composes 30−60% of the polysaccharides found in seaweed. Other carbohydrates include mainly mannitol and glucan, which, unlike alginate, can readily be used in current industry (Enquist-Newman et al. 2014).

Alginates are a family of linear polysaccharides found in brown seaweed and bacteria (Gorin and Spencer 1966*;* Haug et al. 1967). Alginate consists of (1 → 4)-linked β-D-mannuronic acid (M) and its C-5 epimer α-L-guluronic acid (G) arranged in sequences of M, G, or alternating MG blocks (Pawar and Edgar 2012). Alginate is degraded to 4,5-unsaturated monouronates by alginate lyases (Kim et al. 2011). The monouronates are converted to 4-deoxy-L-erythro-hexoseulose uronic acid (DEH), which is reduced to the key metabolite 2-keto-3-deoxygluconate (KDG) by DEH reductase (Preiss and Ashwell 1962). The conversion of monouronates to DEH is catalysed by the enzyme KdgF, a late step of alginate degradation (Hobbs et al. 2016). For understanding the catalytic mechanism and protein dynamics of KdgF further insight is needed.

## Methods and experiments

### Construct design

Genomic DNA was isolated from a culture of *Bacteroides eggerthii* DSM 20,697 purchased from Deutsche Samlung von Mikroorganismen und Zellkulturen (Germany) using Microbiome DNA Purification Kit (Invitrogen) according to manufacturer’s specifications. The gene encoding *Be*KdgF (UniProt: R5JNH6) was amplified using a modified Phusion High-Fidelity DNA polymerase with primers designed for USER cloning (Salomonsen et al. 2014). *Be*KdgF was cloned into the pET15b-USER vector by the restriction sites Ndel and BamHI, which extended *Be*KdgF with an N-terminal His-tag (MGSSHHHHHHGS) resulting in 126 amino acid residues in total, and the full-length construct was used for the resonance assignment. The resulting plasmid (pET15b-USER-*Be*KdgF) was verified by sequencing (GATC Biotech, Germany).

### Sample preparation

For resonance assignment ^15^N–^13^C-labelled *Be*KdgF from *B. eggerthii* was produced by recombinant expression in *Escherichia coli*.

*E. coli* T7 Express cell (New England bioLabs; BL21 (DE3)) transformed with the pET15b-USER-*Be*KdgF plasmid were incubated overnight (37 °C) in a petri dish containing LB medium (10 g/L tryptone, 5 g/L yeast extract, 5 g/L NaCl), agar (1.5%) and ampicillin (100 μg/mL). One colony was transferred to LB medium (25 mL) containing ampicillin (100 μg/mL) and incubated overnight (30 °C, 225 rpm). The preculture (1% v/v) was added to M9 medium (500 mL, 6 g/L Na_2_HPO_4_, 3 g/L KH_2_PO_4_, 0.5 g/L NaCl, 1.0 g/L (^15^NH_4_)_2_SO_4_) along with ampicillin (100 μg/mL), 2 mL MgSO_4_ (1 M), Trace Metal solution (0.1 g/L ZnSO_4_, 0.8 g/L MnSO_4_, 0.5 g/L FeSO_4_, 0.1 g/L CuSO_4_, 1 g/L CaCl_2_), Gibco™ MEM vitamin solution (100x), 10 mL Bioexpress Cell Growth Media (^13^C, ^15^ N labelled) (Cambridge Isotope Laboratories), and 6.0 g/L ^13^C_6_ D-glucose. All isotopes were purchased from Cambridge Isotope Laboratories, Tewksbury, USA. The culture was incubated (30 °C, 225 rpm) until OD_600nm_ was between 0.6 and 0.8, and the culture was then cooled on ice for 5 min. Expression was induced by adding IPTG (isopropyl β-D-1-thiogalactopyranoside) (final conc. 0.5 mM), hereafter the culture was incubated overnight (16 °C, 190 rpm).

The cells were harvested by centrifugation (10 min, 4 °C, 5000×*g*), the supernatant was discarded, and the pellet resuspended in ice cold lysis buffer (50 mM HEPES, 300 mM NaCl, pH 7.7) along with one cOmplete™ EDTA-free protease inhibitor tablet (Roche). The resuspended cells were lysed using sonication for 10 min. using a Branson Sonifier equipped with a microtip. The cell lysate was centrifuged (30 min, 4 °C, 16000×*g*) and the supernatant sterile filtered (0.22 μm filter).

*Be*KdgF was purified with affinity chromatography using Ni^2+^-resin. A column was filled with 3 mL cOmplete His-tag Purification Resin (Roche), and the resin was first rinsed with ethanol (20%, 20 mL) and ultrapure water (20 mL), before being equilibrated with 20 mL lysis buffer. The lysate was loaded onto the column and the column was washed with lysis buffer (3 × 7.5 mL) and washing buffer (50 mM HEPES, 300 mM NaCl, 20 mM imidazole, pH 7.7, 8 × 7.5 mL). The protein was eluded using elution buffer (50 mM HEPES, 300 mM NaCl, 300 mM imidazole, pH 7.7) and stored at 4 °C. The enzyme molecular size and purity were assessed using SDS-PAGE.

The buffer was exchanged to the buffer used for NMR data collection (25 mM Na_2_HPO_4_, 50 mM NaCl, pH 7.2) using a VivaSpin column (5 kDa cut-off, Sartorius) and the sample was then concentrated to a volume of ≈ 130 μL on an Amicon Ultra centrifugal filter (3 kDa cut-off). The concentrated protein sample was transferred to a 3 mm NMR tube and 99.9% D_2_O (10 v/v%) (Sigma Aldrich) was added. The *Be*KdgF concentration in the NMR sample was calculated to 430 μM using an extinction coefficient of 10,095 M^−1^ cm^−1^ (Gasteiger et al. 2005) and measuring absorbance at 280 nm (NanoDrop One Microvolume UV–Vis spectrophotometer, Thermo Fisher Scientific). The N-terminal His-tag did not interfere negatively the NMR work thus removal of the His-tag was not attempted.

### NMR experiments

All NMR spectra were recorded at 20 °C on a Bruker Avance III HD 800 MHz spectrometer using a 5 mm Z-gradient CP-TCI (H/C/N) cryogenic probe at the NV-NMR-Center/Norwegian NMR Platform (NNP) at the Norwegian University of Science and Technology (NTNU). ^1^H signals were internally referenced to the water signal, and ^13^C and ^15^N signals were indirectly referenced to the water signal based on absolute frequency ratios (Zhang et al. 2003).

Backbone and side-chain resonance assignment were accomplished using ^1^H–^15^N HSQC, ^1^H–^13^C HSQC, HNCO, HNcaCO, HNCA, HNCACB. HNcoCACB, HNcoHAHB, HNHA, HcCH-TOCSY, and ^15^ N-NOESY-HSQC with 80 ms mixing time. All spectra were processed using TopSpin version 3.6.1.

Spectra were analysed using CARA (Computer Aided Resonance Assignment) version 1.8.4.2 (Keller 2004). A dihedral angle analysis based on the measured backbone and side-chain chemical shifts was made using TALOS-N (Shen and Bax 2013).

### Assignment and data deposition

**Fig. 1:**
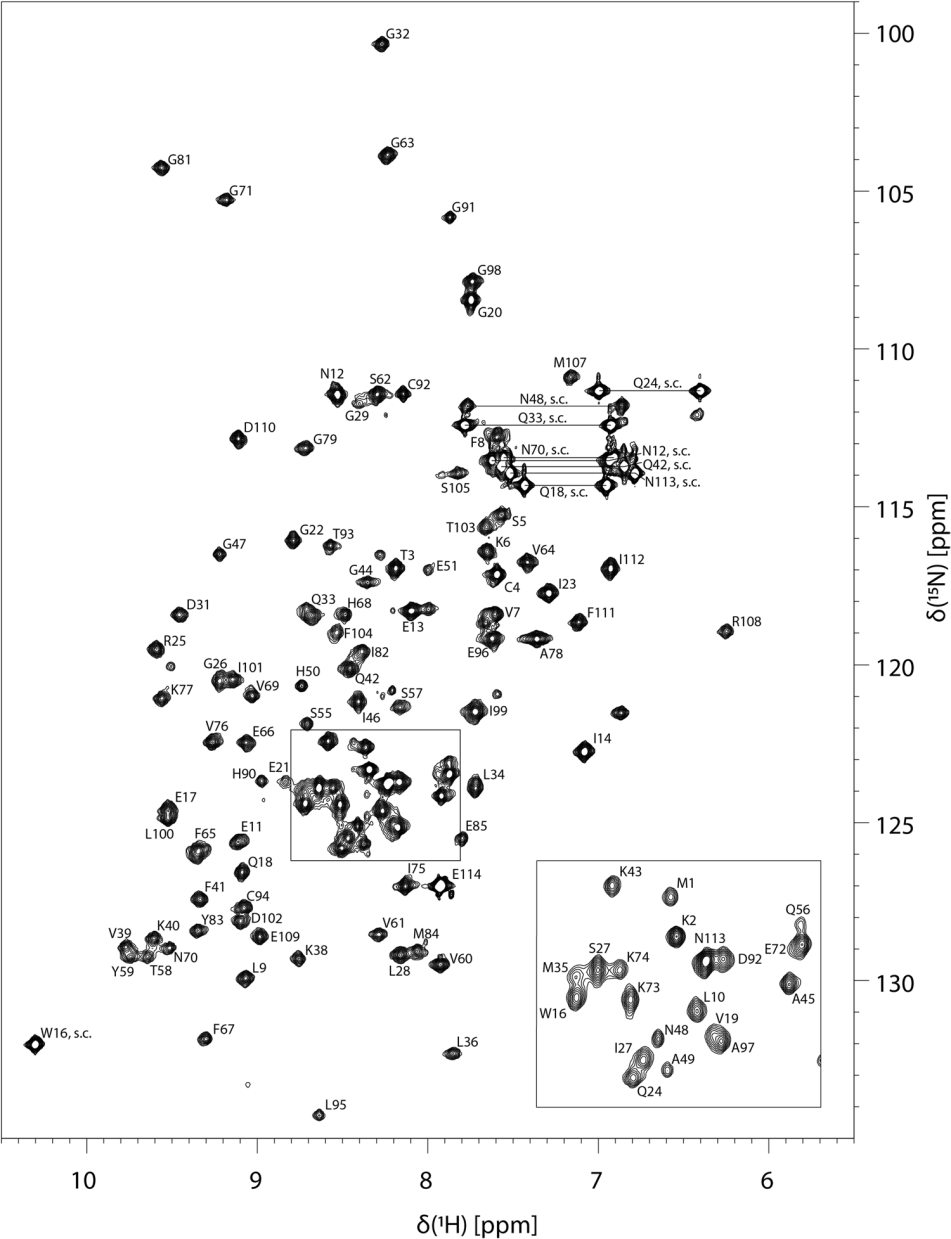
^1^H–^15^N HSQC spectrum of ^13^C–^15^N-labelled *Be*KdgF from *Bacteroides eggerthii* in a NaH_2_PO_4_ buffer at pH 7.2 with 50 mM NaCl in 90% H_2_O/10% D_2_O. Side-chain resonances of Asn and Gln residues are connected by lines. Other side-chain resonances are indicated with the amino acid number and “s.c.”. Residue number and type of backbone and side-chain amide N and H^N^ are indicated

Here we report the backbone and side-chain assignment of *Be*KdgF from *B. eggerthii*. The ^15^N-HSQC spectrum of *Be*KdgF with the assigned resonances is shown in Fig. 1. The backbone assignment is almost complete (H^N^, H^α^, C^α^, N, and C′ > 92%). The five unassigned residues (H52, F53, P86, D87, and V88) define short two sections in *Be*KdgF, which may be ascribed to multiple conformations in intermediate exchange, enhance relaxation or fast exchange regime. The side-chain assignment is partially complete (side-chain H and C ≈ 59.3%). The unassigned smaller peaks in the ^1^H–^15^N HSQC are due to impurities of the sample. The amino acid residue R108 has an unusual chemical shift as the H^N^, C^β^, and H^β^ chemical shifts are significantly lower than expected (Ulrich et al. 2008). The chemical shifts have been deposited in the Biological Magnetic Resonance Data Bank (BMRB) under the accession number 51288.

**Fig. 2:**
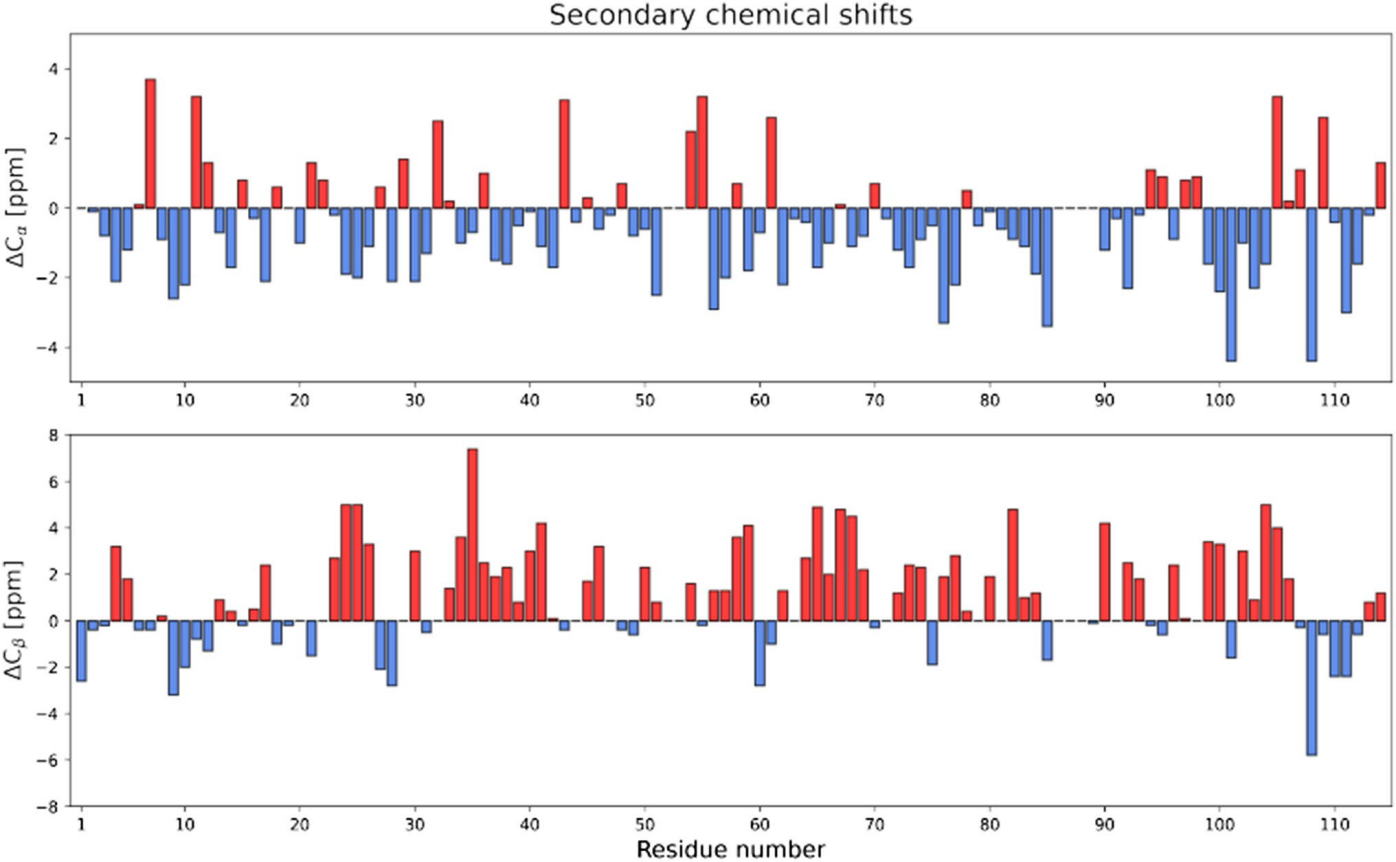
Chemical shift deviation from random-coil values (Wishart et al. 1995) for C_α_ (top) and C_β_ (bottom) of *Be*KdgF

Secondary structural propensity was evaluated by investigating secondary chemical shift values of *Be*KdgF. The chemical shift deviations of C_α_ and C_β_ from the random-coil values for each residue (Wishart et al. 1995) were calculated, and the results can be seen in Fig. 2. Generally, the C_α_ chemical shifts of *Be*KdgF are lower than the predicted random-coil values, whereas the C_β_ values are higher. This indicates that *Be*KdgF generally consists of β-sheets.

**Fig. 3:**
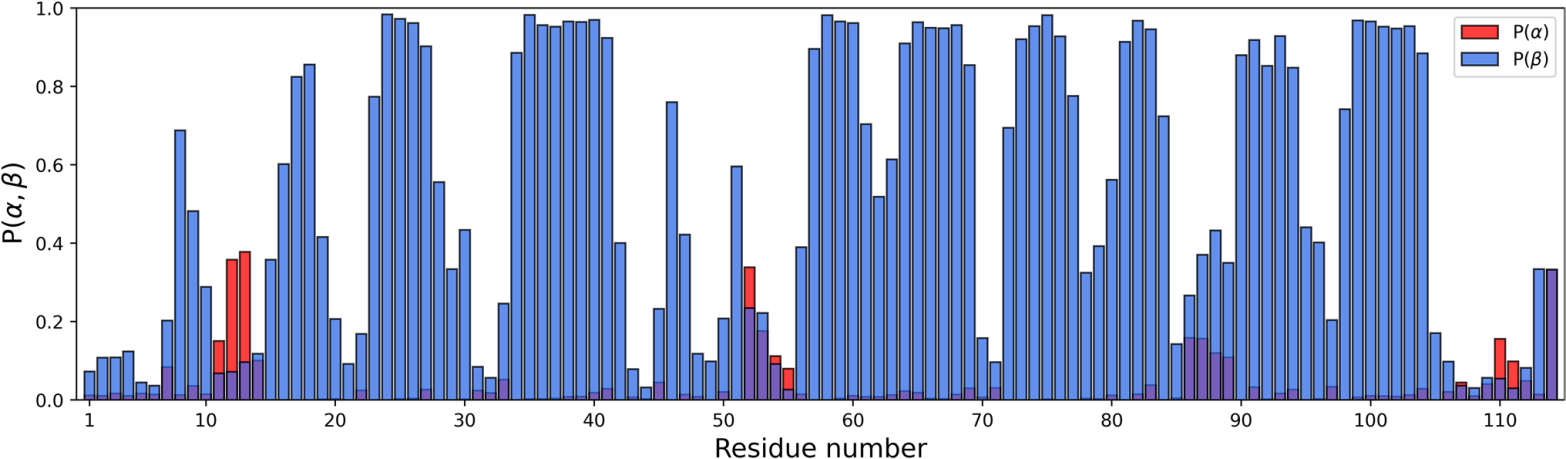
Results from TALONS-N (Shen and Bax 2013). The panel shows the probability of an amino acid residue adopting a specific secondary structure (red = α-helix, blue = β-strand)

The probability of secondary structural elements was calculated based on the dihedral angle analysis using TALOS-N (Shen and Bax, 2013). The results can be seen in Fig. 3. The dihedral angle analysis shows that 50% of the amino acid residues in *Be*KdgF form β-strands and the rest random coils with a possibility of a three amino acid residue α-helix present. The β-sheets content is consistent with the three previously reported structures of KdgF from the organisms *Yersinia enterocolitica* and *Halomonas* sp (Hobbs et al. 2016).

The ^1^H, ^13^C, ^15^N resonance assignment of *Be*KdgF has been presented. The assignment gives the possibility of further investigation of *Be*KdgF with NMR spectroscopy. Future functional studies will include metal ion interactions, pH titration, and protein dynamics. Understanding the biological role of KdgF can aid the industrial use of alginates extracted from seaweed as biomass.

## Data Availability

The assigned chemical shifts have been deposited in the Biological Magnetic Resonance Data Bank (BMRB) under the accession number 51288.
